# Supplementary figures and images for: Targeting prolyl-tRNA synthetase via a series of ATP-mimetics to accelerate drug discovery against toxoplasmosis
Source: PLoS Pathog. 2023 Feb 28;19(2):e1011124. doi: 10.1371/journal.ppat.1011124 (PMC9974123; doi:10.1371/journal.ppat.1011124)

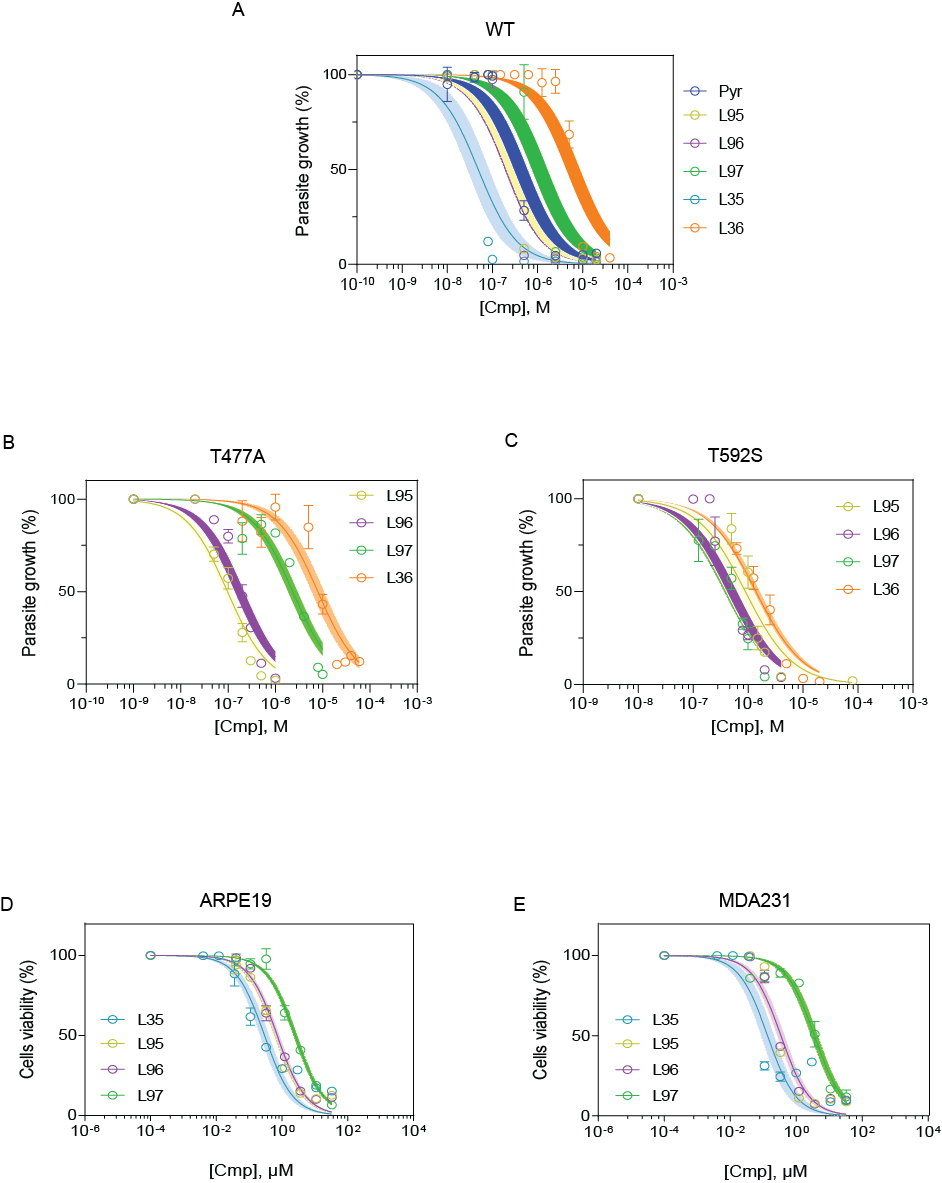

Supplement: S1 Fig — A-C) Graphs showing the dose-response curves of T. gondii parasites in the presence of the MMV compounds indicated. Confluent HFFs were infected with wild-type (A), T447A (B) or T592S (C) edited parasites expressing the Nanoluc luciferase. After 48h of incubation, parasite proliferation was quantified to calculate the IC50 by non-linear regression analysis. The graphs represent the mean ±SD of 3 technical replicates from one experiment. Shaded error envelopes depict 95% confidence intervals. D-E) Dose-response curves of ARPE-19 and MDA231 cell lines in presence of different MMV drugs. Human cells were plated on 96 wells plates and incubated with growing concentrations of drugs. After 72h, the cells’ viability was revealed using the CellTiter-Blue assay kit (Promega) and the CC50 was calculated. The graphs represent the mean ± SD of 3 technical replicates from one experiment. (TIF) [file ppat.1011124.s001.tif]

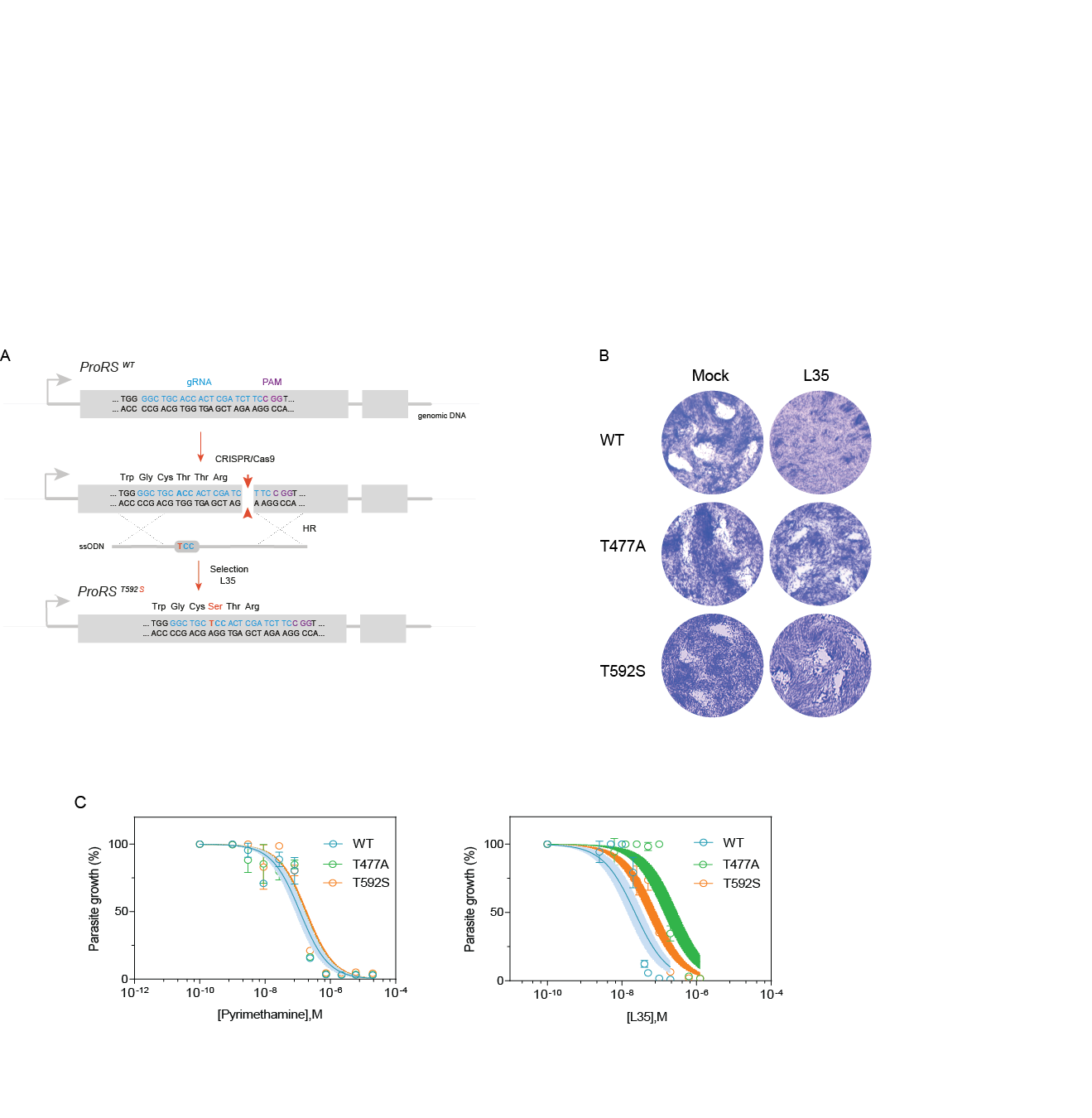

Supplement: S2 Fig — A) Schematic of PRS gene editing strategy to introduce point mutations in T. gondii parasites. Focus on the PRS locus and CRISPR/Cas9-mediated homology-directed repair with single-stranded oligo DNA nucleotides (ssODNs) carrying nucleotide substitutions (red letters). After homologous recombination (HR) events, PRS recombinant parasites were selected with L35. Only T592S is shown for clarity. B) Images of plaques formed by recombinant parasites after 7 days in presence of 0.1μM of L35 or 0.1%DMSO. C) Dose-response curve of T477A and T592S edited parasites incubated with increasing concentration of Pyrimethamine or L35. The graphs represent one of three different experiments. The mean ±SD is from 3 technical replicates of one assay and the shaded error envelopes depict 95% confidence intervals. (TIF) [file ppat.1011124.s002.tif]

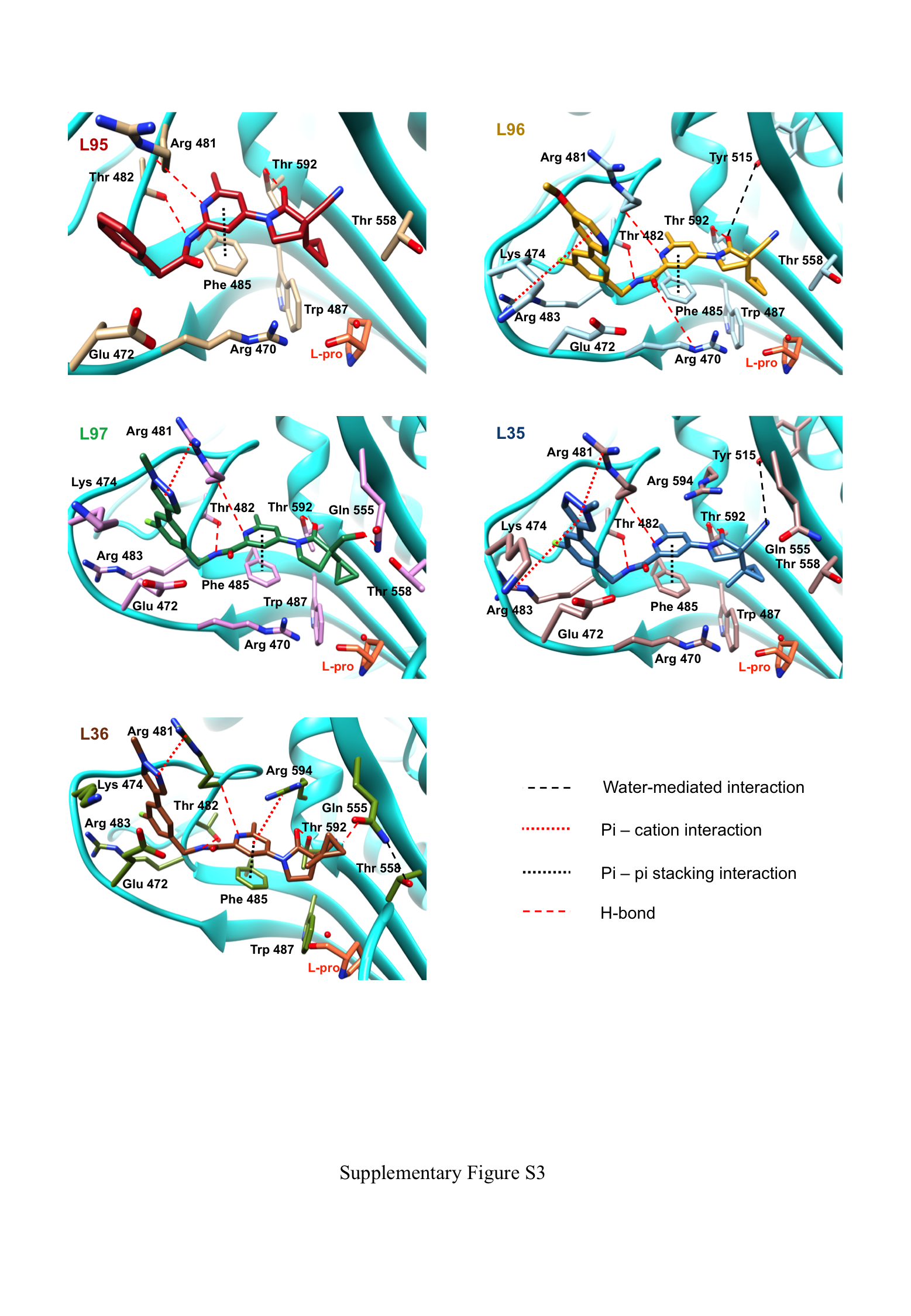

Supplement: S3 Fig — The structural interactions associated with (A) L95, (B) L96, (C) L97, (D) L35, (E) L36 in TgPRS active site are shown. (TIF) [file ppat.1011124.s003.tif]

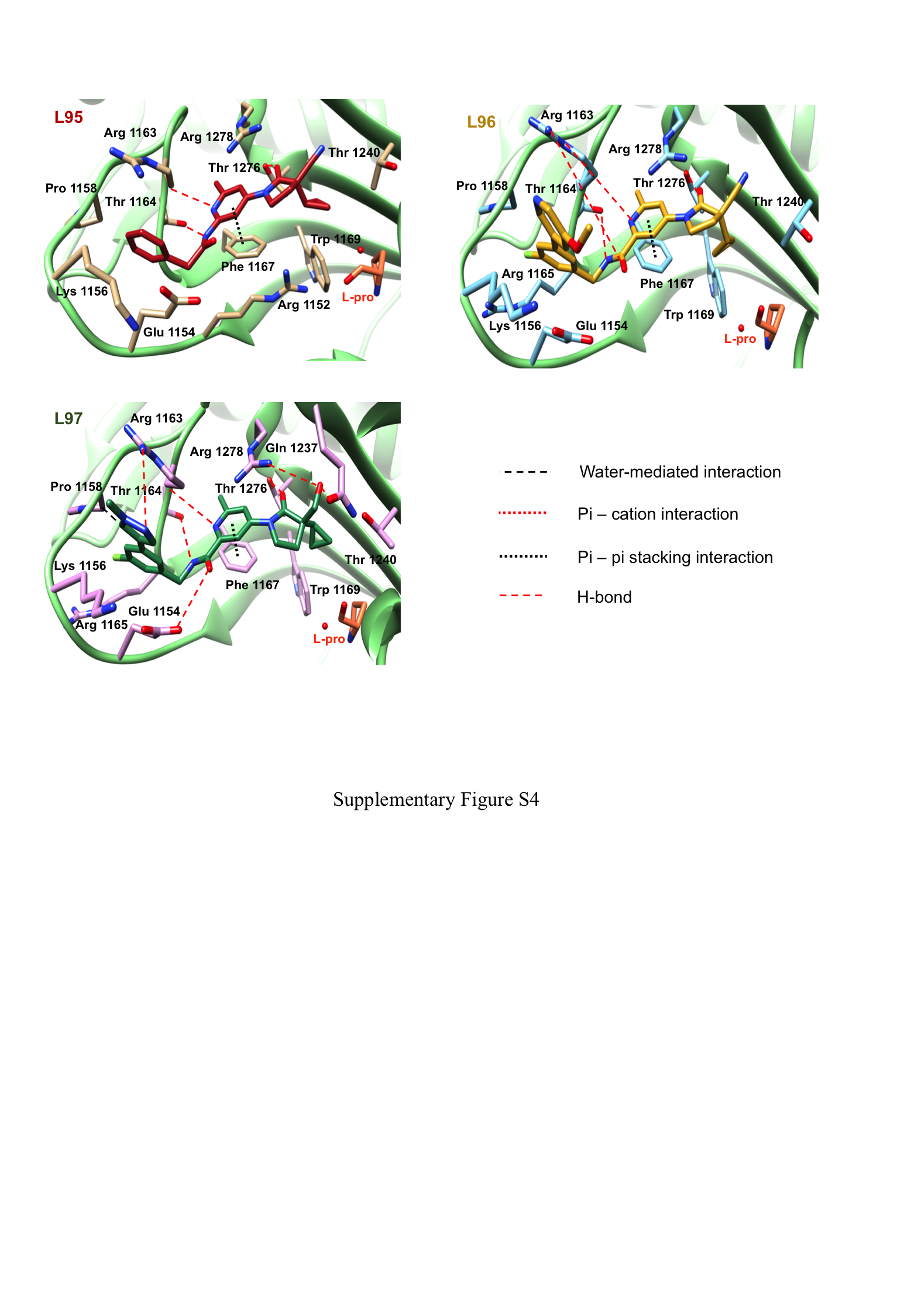

Supplement: S4 Fig — The structural interactions associated with (A) L95, (B) L96, (C) L97 in HsPRS active site are shown. (TIF) [file ppat.1011124.s004.tif]
