# Supplementary material for: Targeting prolyl-tRNA synthetase via a series of ATP-mimetics to accelerate drug discovery against toxoplasmosis
Source: PLoS Pathog. 2023 Feb 28;19(2):e1011124. doi: 10.1371/journal.ppat.1011124 (PMC9974123; doi:10.1371/journal.ppat.1011124)
Supplement: S1 Table — The varying alleles of the resistant mutants found in candidate gene PRS are displayed. Amino-acid substitutions with the corresponding codons shown in parentheses are indicated for each mutagenized T.gondii L35-resistant strain. (DOC) [file ppat.1011124.s005.doc]

**Supplementary Table S1** Mutations found in candidate genes by RNA-Sequencing analysis of L35-resistant mutants

|  |  |  |  | Variant calling | | | | | | |
| --- | --- | --- | --- | --- | --- | --- | --- | --- | --- | --- |
|  |  |  |  | Parental strain | Resistant mutants | | | | | |
| Chr. | Gene | Annotation | Position | WT | 35-1 | 35-2 | 35-3 | 35-4 | 35-5 | 35-6 |
| XII | TGGT1_219850 | *Tg*PRS | 533178 |  | T477A (ACG to GCG) | T477A (ACG to GCG) | T477A (ACG to GCG) | T477A (ACG to GCG) |  | T477A (ACG to GCG) |
| XII | TGGT1_219850 | *Tg*PRS | 531556 |  |  |  |  |  | T592S (ACC to TCC) |  |

Amino acid substitutions with the corresponding codons shown in parentheses are indicated for each mutagenized *T. gondii*-resistant mutant strain.
