# Supplementary material for: Targeting prolyl-tRNA synthetase via a series of ATP-mimetics to accelerate drug discovery against toxoplasmosis
Source: PLoS Pathog. 2023 Feb 28;19(2):e1011124. doi: 10.1371/journal.ppat.1011124 (PMC9974123; doi:10.1371/journal.ppat.1011124)
Supplement: S2 Table — (DOC) [file ppat.1011124.s006.doc]

| **Name** | **PDB ID** | **Growth condition** |
| --- | --- | --- |
| *Tg*PRS-L95 | 7EVV | 0.1 M Sodium cacodylate pH 6.5, 40% v/v MPD, 5% w/v PEG 8000 |
| *Tg*PRS-L95 | 7VC1 | 0.1 M Amino acids^#a1^, 0.1 M Buffer^#b1^ pH 6.5, 30% v/v Precipitant ^#p1^ |
| *Tg*PRS-L96 | 7FAK | 0.1 M Carboxylic acids^#2^, 0.1 M Buffer^#b1^ pH 6.5 and 30% v/v Precipitant ^#p1^ |
| *Tg*PRS-L96 | 7VC2 | 0.12 M Ethylene glycols^#a3^, 0.1 M Buffer^#b1^ pH 6.5, 30% v/v Precipitant ^#p1^ |
| *Tg*PRS-L97 | 7FAM | 0.1 M Amino acids^#a1^, 0.1 M Buffer^#b1^ pH 6.5, 30 % v/v Precipitant ^#p2^ |
| *Tg*PRS-L97 | 7VC3 | 0.1 M Amino acids^#a1^, 0.1 M Buffer^#b2^ pH 7.5, 30% v/v Precipitant ^#p2^ |
| *Tg*PRS-L35 | 7FAN | 0.1 M Amino acids^#a1^, 0.1 M Buffer^#b1^ pH 6.5, 30% v/v Precipitant ^#p1^ |
| *Tg*PRS-L36 | 7FAL | 0.12 M Ethylene glycols^#a3^, 0.1 M Buffer^#b1^ pH 6.5, 30 % v/v Precipitant ^#p1^ |
| *Hs*PRS-L95 | 7F9B | 0.1 M HEPES pH 7.5, 1.5 M CaCl2 and 20% PEG 3350 |
| *Hs*PRS-L95 | 7F98 | 0.1 M HEPES pH 7.5, 0.5 M CaCl2 and 20% PEG3350 |
| *Hs*PRS-L96 | 7F9C | 0.1 M HEPES pH 7.5, 0.5 M CaCl2 and 20% PEG3350 |
| *Hs*PRS-L96 | 7F99 | 0.1 M HEPES pH 7.5, 1.5 M CaCl2 and 20% PEG3350 |
| *Hs*PRS-L96 | 7F9D | 0.1 M HEPES pH 7.5, 0.5 M CaCl2 and 20% PEG3350 |
| *Hs*PRS-L97 | 7F9A | 0.1 M HEPES pH 7.5, 0.5 M CaCl2 and 20% PEG3350 |

**Supplementary Table S2** Growth conditions for PRS crystals.

^#a1^ 0.2 M DL-Glutamic acid monohydrate, 0.2 M DL-Alanine, 0.2 M Glycine, 0.2 M DL-Lysine monohydrochloride and 0.2 M DL-Serine

^#a2^ 0.2 M Sodium formate; 0.2 M Ammonium acetate; 0.2 M Sodium citrate tribasic dihydrate; 0.2 M Potassium sodium tartrate tetrahydrate; 0.2 M Sodium oxamate

^#a3^ (0.3 M Diethylene glycol, 0.3 M Triethylene glycol, 0.3 M Tetraethylene glycol, 0.3 M Pentaethylene glycol)

^#a4^ 0.2 M 1,6-Hexanediol, 0.2 M 1-Butanol, 0.2 M 1,2-Propanediol, 0.2 M 2-Propanol, 0.2 M 1,4-Butanediol, 0.2 M 1,3-Propanediol

^#b1^ Imidazole; MES monohydrate (acid)

^#b2^ Sodium HEPES; MOPS (acid)

^#b3^ Tris (base); BICINE

^#p1^ (40% v/v Ethylene glycol, 20% w/v PEG 8000)

^#p2^ 40% v/v Glycerol; 20% w/v PEG 4000

^#p3^ 25% v/v MPD, 25% PEG 1000, 25% w/v PEG 3350
