# Supplementary material for: Targeting prolyl-tRNA synthetase via a series of ATP-mimetics to accelerate drug discovery against toxoplasmosis
Source: PLoS Pathog. 2023 Feb 28;19(2):e1011124. doi: 10.1371/journal.ppat.1011124 (PMC9974123; doi:10.1371/journal.ppat.1011124)
Supplement: S3 Table — (DOC) [file ppat.1011124.s007.doc]

**Supplementary Table S3.** Summary PDB entries associated with this manuscript.

| **Name** | **PDB ID** | **SG** | **Res. (Å)** | **R_w_/R_f_ (%)** | **B@ (Å^2^)** | **CS^** | **Rama* (%)** |
| --- | --- | --- | --- | --- | --- | --- | --- |
| *Tg*PRS-L95 | 7EVV | C2 | 1.70 | 15.3/18.1 | 32 | 3 | 98.5/1.3/0.2 |
| *Tg*PRS-L95 | 7VC1 | P2_1_ | 1.89 | 17.4/21.1 | 40 | 3 | 98.4/1.5/0.1 |
| *Tg*PRS-L96 | 7FAK | C2 | 1.90 | 17.6/20.5 | 43 | 6 | 97.9/2.1/0.0 |
| *Tg*PRS-L96 | 7VC2 | P2_1_ | 2.10 | 17.7/21.8 | 51 | 5 | 98.1/1.7/0.2 |
| *Tg*PRS-L97 | 7FAM | C2 | 2.42 | 18.2/23.3 | 56 | 5 | 97.3/2.5/0.2 |
| *Tg*PRS-L97 | 7VC3 | C2 | 1.97 | 17.5/21.7 | 54 | 3 | 98.1/1.7/0.2 |
| *Tg*PRS-T35 | 7FAN | C2 | 1.77 | 17.5/21.8 | 42 | 4 | 97.7/2.1/0.2 |
| *Tg*PRS-T36 | 7FAL | P2_1_ | 3.22 | 22.9/25.3 | 53 | 8 | 96.4/3.2/0.4 |
| *Hs*PRS-L95 | 7F9B | P2_1_ | 2.0 | 18.9/23.6 | 47 | 4 | 98.5/1.3/0.2 |
| *Hs*PRS-L95 | 7F98 | P2_1_2_1_2_1_ | 2.0 | 19.0/23.5 | 53 | 5 | 98.4/1.4/0.2 |
| *Hs*PRS-L96 | 7F9C | P2_1_ | 2.20 | 18.8/25.3 | 57 | 5 | 97.7/2.1/0.2 |
| *Hs*PRS-L96 | 7F99 | P2_1_ | 1.99 | 18.1/22.8 | 43 | 3 | 98.4/1.4/0.2 |
| *Hs*PRS-L96 | 7F9D | P2_1_2_1_2_1_ | 2.5 | 18.7/26.3 | 54 | 5 | 98.7/1.0/0.3 |
| *Hs*PRS-L97 | 7F9A | P2_1_2_1_2_1_ | 2.0 | 19.5/24.2 | 61 | 4 | 98.4/1.3/0.3 |

SG - Space group, Res. - Resolution, Fo,Fc Cor - Fo,Fc correlation, B@ (Å2) - Average B for all atoms, CS^ -Clashscore, Rama* (%) - Ramachandran plot Favoured/Allowed/Outliers
