# Supplementary material for: Targeting prolyl-tRNA synthetase via a series of ATP-mimetics to accelerate drug discovery against toxoplasmosis
Source: PLoS Pathog. 2023 Feb 28;19(2):e1011124. doi: 10.1371/journal.ppat.1011124 (PMC9974123; doi:10.1371/journal.ppat.1011124)
Supplement: S4 Table — Statistics for the highest-resolution shell are shown in parenthesis. (DOC) [file ppat.1011124.s008.doc]

**Supplementary Table S4.** Summary of data collection and refinement statistics. Statistics for the highest-resolution shell are shown in parentheses.

| **PRS-Inhibitor** | **TgPRS-L95^!^** | **TgPRS-L95** | **TgPRS-L96** |
| --- | --- | --- | --- |
| PDB code | 7EVV | 7VC1 | 7FAK |
| **Data collection and processing** |  |  |  |
| Diffraction source | PROXIMA-1 (PX1) | PROXIMA-1 (PX1) | DLS I04 |
| Wavelength (Å) | 0.979 | 0.9786 | 0.9795 |
| Detector | EIGER X 16M | EIGER X 16M |  |
| Total rotation range (°) | 360 | 360 | 720 |
| Space group | C2 | P2_1_ | C2 |
| a, b, c (Å) | 111.35, 75.20, 74.74 | 76.89, 81.80, 104.66 | 113.62, 74.23, 74.65 |
| α, β, γ (°) | 90.0, 110.7, 90.0 | 90.0, 102.6, 90.0 | 90.0, 111.1, 90.0 |
| Resolution (Å) | 41.85–1.70 (1.71–1.70) | 47.29–1.89 (2.01–1.89) | 60.81–1.89 (1.94–1.89) |
| No. of unique reflections | 62494 (9672) | 100749 (15940) | 45141 (2157) |
| Completeness (%) | 99.1 (95.7) | 99.6 (97.9) | 98.6 (94.9) |
| Redundancy | 2.2 (2.3) | 7.0 (6.7) | 6.6 (5.7) |
| I/σI | 13.3 (2.9) | 10.1 (1.5) | 8.7 (1.3) |
| Half-set correlation CC_1/2_ ^*^ | 1.0 (0.8) | 1.0 (0.8) | 1.0 (0.5) |
| R_meas_^**^ | 0.089 (6.02) | 0.118 (1.163) | 0.125 (0.890) |
| Wilson B-factor (Å^2^) | 26.2 | 33.0 | 26.2 |
| **Structure refinement** |  |  |  |
| Resolution (Å) | 31.52–1.70 | 47.29–1.89 | 60.84–1.89 |
| No. of reﬂections /test set | 62474 / 3122 | 100697 / 5035 | 41502 / 2199 |
| R_work_/ R_free_ (%) | 15.3 / 18.1 | 17.4 / 21.1 | 18.1 / 21.6 |
| No. of molecules in ASU | 1 | 2 | 1 |
| No. of protein residues | 490 | 962 | 483 |
| No. of protein atoms/water molecules | 4040 / 287 | 7794 / 628 | 3928 / 286 |
| No. of ligand atoms | 36 + 8 | 36 + 8 | 37 + 8 |
| No. of ions^#^ | 1 | 4 | - |
| No. of solvent atoms^$^ | 16 | 52 | 52 |
| **R.m.s deviations** |  |  |  |
| Bond lengths (Å) | 0.018 | 0.007 | 0.016 |
| Bond angles (º) | 1.508 | 0.895 | 1.309 |
| **Average B factors (Å^2^)** |  |  |  |
| Protein / water | 31.6 / 37.9 | 39.8 / 48.1 | 30.8 / 37.82 |
| Ligand atoms / ions | 21.7 / 77.4 | 27.4 / 69.9 | 35.33 / - |
| **Ramachandran plot** |  |  |  |
| Favoured / Allowed / Outliers (%) | 98.5 / 1.3 / 0.2 | 98.4 / 1.5 / 0.1 | 97.9 / 2.1 / 0.0 |

| **PRS-Inhibitor** | **TgPRS-L96** | **TgPRS-L97** | **TgPRS-L97** |
| --- | --- | --- | --- |
| PDB code | 7VC2 | 7FAM | 7VC3 |
| **Data collection and processing** |  |  |  |
| Diffraction source | PROXIMA 2 | PROXIMA 2 | DLS I24 |
| Wavelength (Å) | 0.9804 | 0.9804 | 0.9686 |
| Detector | EIGER X 9M | EIGER X 9M |  |
| Total rotation range (°) | 360 | 360 | 360 |
| Space group | P2_1_ | C2 | C2 |
| a, b, c (Å) | 77.10, 81.62, 104.88 | 115.49, 74.04, 74.85 | 114.73, 73.64, 74.91 |
| α, β, γ (°) | 90.0, 102.68, 90.0 | 90.0, 111.24, 90.0 | 90.0, 111.38, 90.0 |
| Resolution (Å) | 47.42–2.10 (2.23–2.10) | 52.87–2.42 (2.48–2.42) | 60.64–1.97 (2.01–1.97) |
| No. of unique reflections | 74227 (11607) | 22556 (3400) | 40410 (2008) |
| Completeness (%) | 99.4 (96.9) | 100 (91.0) | 98.6 (97.9) |
| Redundancy | 6.9 | 6.8 | 6.9 (7.1) |
| I/σI | 14.3 (1.7) | 7.4 (1.4) | 10.7 (0.6) |
| Half-set correlation CC_1/2_ | 1.0 (0.7) | 1.0 (0.9) | 1.0 (0.4) |
| R_meas_ | 0.091 (0.977) | 0.092 (0.993) | 0.109 (4.191) |
| Wilson B-factor (Å^2^) | 43.2 | 48.7 | 44.2 |
| **Structure refinement** |  |  |  |
| Resolution (Å) | 47.42–2.10 | 53.87–2.42 | 52.68–1.97 |
| No. of reﬂections /test set | 145446 / 7280 | 22556 / 1076 | 39532 / 1981 |
| R_work_/ R_free_ (%) | 17.7 / 21.8 | 18.2 / 23.3 | 17.5 / 21.7 |
| No. of molecules in ASU | 2 | 1 | 1 |
| No. of protein residues | 947 | 480 | 483 |
| No. of protein atoms/water molecules | 7654 / 416 | 3916 / 47 | 3911 / 130 |
| No. of ligand atoms | 90 | 43 | 43 |
| No. of ions^#^ | 1 | - | 12 |
| No. of solvent atoms^$^ | 48 | - | 26 |
| **R.m.s deviations** |  |  |  |
| Bond lengths (Å) | 0.008 | 0.009 | 0.008 |
| Bond angles (º) | 0.964 | 0.972 | 0.928 |
| **Average B factors (Å^2^)** |  |  |  |
| Protein / water | 51.0 / 54.1 | 57.6 / 51.2 | 53.6 / 55.1 |
| Ligand atoms / ions | 54.5 / 85.4 | 49.9 / - | 42.7 / 91.4 |
| **Ramachandran plot** |  |  |  |
| Favoured / Allowed / Outliers (%) | 98.1 / 1.7 / 0.2 | 97.3 / 2.5 / 0.2 | 98.1 / 1.7 / 0.2 |

| **PRS-Inhibitor** | **TgPRS-L35** | **TgPRS-L36** | **HsPRS-L95** |
| --- | --- | --- | --- |
| PDB code | 7FAN | 7FAL | 7F9B |
| **Data collection and processing** |  |  |  |
| Diffraction source | PROXIMA-1 (PX1) | DLS I04 | PROXIMA 2 |
| Wavelength (Å) | 0.9786 | 0.9795 | 0.9799 |
| Detector | EIGER X 16M |  | EIGER X 9M |
| Total rotation range (°) | 360 | 720 | 360 |
| Space group | C2 | P2_1_ | P2_1_ |
| a, b, c (Å) | 114.52, 74.30, 75.07 | 76.01. 86.33, 104.46 | 70.73, 90.96, 83.49 |
| α, β, γ (°) | 90.0, 111.2, 90.0 | 90.0, 102.33, 90.0 | 90.0, 110.03, 90.0 |
| Resolution (Å) | 52,65–1.77 (1.82–1.77) | 67.25–3.22 (3.33–3.22) | 45.48–2.0 (2.11–2.0) |
| No. of unique reflections | 57267 (4208) | 22703 (1124) | 66905 (10359) |
| Completeness (%) | 100 (100) | 99.9 (100) | 98.4 (94.7) |
| Redundancy | 6.5 (5.9) | 6.9 (6.9) | 6.9 |
| I/σI | 9.2 (1.2) | 4.8 (1.1) | 9.9 (1.1) |
| Half-set correlation CC_1/2_ (%) | 1.0 (0.5) | 1.0 (0.5) | 1.0 (0.4) |
| R_meas_ | 0.091 (1.301) | 0.480 (2.230) | 0.146 (1.553) |
| Wilson B-factor (Å^2^) | 30.1 | 50.4 | 40.0 |
| **Structure refinement** |  |  |  |
| Resolution (Å) | 36.53–1.77 | 54.74–3.22 | 45.48–2.0 |
| No. of reﬂections /test set | 57207 / 2863 | 21485 / 1009 | 66490 / 3328 |
| R_work_/ R_free_ (%) | 17.5 / 21.8 | 23.3 / 25.4 | 18.9 / 23.6 |
| No. of molecules in ASU | 1 | 2 | 2 |
| No. of protein residues | 486 | 958 | 968 |
| No. of protein atoms/water molecules | 3936 / 209 | 7816 / 7 | 7597 / 266 |
| No. of ligand atoms | 44 | 88 | 72 |
| No. of ions^#^ | - | - | 11 |
| No. of solvent atoms^$^ | - | - | - |
| **R.m.s deviations** |  |  |  |
| Bond lengths (Å) | 0.017 | 0.005 | 0.008 |
| Bond angles (º) | 1.444 | 0.967 | 0.965 |
| **Average B factors (Å^2^)** |  |  |  |
| Protein / water | 41.6 / 45.2 | 52.7 / 21.1 | 46.9 / 48.3 |
| Ligand atoms / ions | 31.5 / - | 56.4 / - | 35.5 / 66.1 |
| **Ramachandran plot** |  |  |  |
| Favoured / Allowed / Outliers (%) | 97.7 / 2.1 / 0.2 | 96.4 / 3.2 / 0.4 | 98.5 / 1.3 / 0.2 |

| **PRS-Inhibitor** | **HsPRS-L95** | **HsPRS-L96** | **HsPRS-L96** |
| --- | --- | --- | --- |
| PDB code | 7F98 | 7F9C | 7F99 |
| **Data collection and processing** |  |  |  |
| Diffraction source | PROXIMA 2 | PROXIMA 2 | PROXIMA 2 |
| Wavelength (Å) | 0.9801 | 0.9801 | 0.9801 |
| Detector | EIGER X 9M | EIGER X 9M | EIGER X 9M |
| Total rotation range (°) | 360 | 360 | 360 |
| Space group | P2_1_2_1_2_1_ | P2_1_ | P2_1_ |
| a, b, c (Å) | 70.72, 196.11, 145.29 | 70.72, 196.11, 145.29 | 70.47, 91.52, 83.34 |
| α, β, γ (°) | 90.0, 90.0, 90.0 | 90.0, 90.0, 90.0 | 90.0, 110.11, 90.0 |
| Resolution (Å) | 59.94–2.0 (2.12–2.0) | 62.29–2.20 (2.33–2.20) | 78.25–1.98 (2.01–1.98) |
| No. of unique reflections | 74529 (11755) | 50785 (8075) | 69229 (3404) |
| Completeness (%) | 99.7 (98.4) | 99.6 (98.8) | 99.9 (98.4) |
| Redundancy | 13.4 | 6.2 | 6.7 |
| I/σI | 12.6 (0.8) | 7.3 (0.5) | 9.5 (2.0) |
| Half-set correlation CC_1/2_ | 1.0 (0.5) | 1.0 (0.4) | 1.0 (0.5) |
| R_meas_ | 0.13(2.65) | 0.18 (2.22) | 0.12 (0.854) |
| Wilson B-factor (Å^2^) | 46.8 | 55.3 | 31.7 |
| **Structure refinement** |  |  |  |
| Resolution (Å) | 59.94–2.0 | 62.29–2.20 | 78.25–1.98 |
| No. of reﬂections /test set | 74444 / 3722 | 99589 / 4976^&^ | 69187 / 3366 |
| R_work_/ R_free_ (%) | 19.0 / 23.5 | 18.8 / 25.3 | 18.1 / 22.8 |
| No. of molecules in ASU | 2 | 2 | 2 |
| No. of protein residues | 983 | 974 | 972 |
| No. of protein atoms/water molecules | 7801 / 296 | 7709 / 173 | 7011 / 407 |
| No. of ligand atoms | 72 | 90 | 90 |
| No. of ions^#^ | 12 | 13 | 9 |
| No. of solvent atoms^$^ | - | - | - |
| **R.m.s deviations** |  |  |  |
| Bond lengths (Å) | 0.008 | 0.008 | 0.007 |
| Bond angles (º) | 0.958 | 0.998 | 0.929 |
| **Average B factors (Å^2^)** |  |  |  |
| Protein / water | 53.9 / 53.6 | 57.2 / 52.65 | 42.9 / 44.8 |
| Ligand atoms / ions | 41.9 / 83.6 | 48.6 / 70.3 | 27.2 / 62.7 |
| **Ramachandran plot** |  |  |  |
| Favoured / Allowed / Outliers (%) | 98.4 / 1.4 / 0.2 | 97.7 / 2.1 / 0.2 | 98.4 / 1.4 / 0.2 |

| **PRS-Inhibitor** | **HsPRS-L96** | **HsPRS-L97** |
| --- | --- | --- |
| PDB code | 7F9D | 7F9A |
| **Data collection and processing** |  |  |
| Diffraction source | PROXIMA 2 | PROXIMA 2 |
| Wavelength (Å) | 0.980 | 0.9801 |
| Detector | EIGER X 9M | EIGER X 9M |
| Total rotation range (°) | 360 | 360 |
| Space group | P2_1_2_1_2_1_ | P2_1_ |
| a, b, c (Å) | 70.49, 106.13, 145.55 | 70.72, 196.11, 145.29 |
| α, β, γ (°) | 90.0, 90.0, 90.0 | 90.0, 90.0, 90.0 |
| Resolution (Å) | 44.13-2.50 (2.65-2.50) | 59.93-2.0 (2.12-2.0) |
| No. of unique reflections | 38588 (6065) | 74577 (11817) |
| Completeness (%) | 99.7 (98.5) | 99.8 (98.9) |
| Redundancy | 13.2 | 15.0 |
| I/σI | 9.2 (1.5) | 12.5 (0.5) |
| Half-set correlation CC_1/2_ | 1.0 (0.6) | 1.0 (0.5) |
| R_meas_ | 0.24 (1.50) | 0.13 (2.61) |
| Wilson B-factor (Å^2^) | 52.4 | 53.3 |
| **Structure refinement** |  |  |
| Resolution (Å) | 44.13-2.50 | 59.93-2.0 |
| No. of reﬂections /test set | 73020 / 3650^&^ | 74344 / 3721 |
| R_work_/ R_free_ (%) | 18.7 / 26.3 | 19.5 / 24.2 |
| No. of molecules in ASU | 2 | 2 |
| No. of protein residues | 980 | 987 |
| No. of protein atoms/water molecules | 7767 / 113 | 7787 / 236 |
| No. of ligand atoms | 90 | 86 |
| No. of ions^#^ | 12 | 15 |
| No. of solvent atoms^$^ | - | - |
| **R.m.s deviations** |  |  |
| Bond lengths (Å) | 0.009 | 0.008 |
| Bond angles (º) | 1.033 | 0.946 |
| **Average B factors (Å^2^)** |  |  |
| Protein / water | 54.4 / 50.0 | 61.9 / 58.9 |
| Ligand atoms / ions | 54.4 / 78.5 | 48.0 / 83.1 |
| **Ramachandran plot** |  |  |
| Favoured / Allowed / Outliers (%) | 98.7 / 1.0 / 0.3 | 98.4 / 1.3 / 0.3 |
