# Supplementary material for: Targeting prolyl-tRNA synthetase via a series of ATP-mimetics to accelerate drug discovery against toxoplasmosis
Source: PLoS Pathog. 2023 Feb 28;19(2):e1011124. doi: 10.1371/journal.ppat.1011124 (PMC9974123; doi:10.1371/journal.ppat.1011124)
Supplement: S5 Table — (DOC) [file ppat.1011124.s009.doc]

**Table S5 Summary of EC50 values of WT and mutants against L35.**

| **Mutant Parasite** | **EC_50_ Treated-PYR (nM)** | **EC_50_ Treated-L95 (nM)** | **EC_50_ Treated-L96 (nM)** | **EC_50_ Treated-L97 (nM)** | **EC_50_ Treated-L35 (nM)** | **EC_50_ Treated-L36 (nM)** |
| --- | --- | --- | --- | --- | --- | --- |
| WT | 174 ± 60 | 277 ± 6 | 106 ± 36 | 544 ± 130 | 31 ± 12 | 5800 ± 350 |
| T477A | 198 ± 70 | 174 ± 120 | 249 ± 110 | 1800 ± 410 | 186 ± 12 | 7800 ± 700 |
| T592S | 241 ± 140 | 1000 ± 170 | 525 ± 7 | 470 ± 28 | 76 ± 19 | 1600 ± 310 |
