# Supplementary material for: Targeting prolyl-tRNA synthetase via a series of ATP-mimetics to accelerate drug discovery against toxoplasmosis
Source: PLoS Pathog. 2023 Feb 28;19(2):e1011124. doi: 10.1371/journal.ppat.1011124 (PMC9974123; doi:10.1371/journal.ppat.1011124)
Supplement: Attachment — Submitted filename: Rev-Tg-Tak5_Response_Reviewers_221222.docx [file ppat.1011124.s011.docx]

**Part I – Summary**

**Please use this section to discuss strengths/weaknesses of study, novelty/significance, general execution and scholarship.**

**Reviewer #1:** In the study by Yogavel et al., a thorough inhibitor study on Toxoplasma gondii and human prolyl-tRNA synthetase (PRS) was performed, using an interdisciplinary approach, involving chemical mutagenesis and reverse genetics, biochemical and structural analysis. PRS has been previously described as a promising drug target and similar biochemical studies (using different inhibitors) have been previously described by the same groups. Here, 5 compounds based on the ATP-mimetics 1-(pyridin-4-yl) pyrrolidin-2-one (PPL), named L95, L96, L97, L35 and L36 were investigated in great detail. Briefly, the authors found that L35 shows the highest selective inhibition by comparing toxicity on the parasite and its host cells. Next, chemical mutagenesis was performed and 2 mutants (T477A and T592A) in the coding region of TgPRS were identified to confer resistance to L35 and other PPL derivatives, clearly demonstrating that PRS is the target. Using well established biochemical assays, inhibition of human and TgPRS was compared using these inhibitors and the in vitro data nicely reflect the findings in vivo.
Finally, the authors succeed in solving and comparing several structures of human and parasite PRS bound to inhibitors and L-Pro. In summary, this well performed study opens an avenue for future structure-based optimisation of PRS inhibitors that appear to be very promising for future intervention strategies.
Since this reviewer has no significantl experience in structural biology, minor concerns are only on the remaining experiments.

**Reply:** We thank the reviewer for their astute and brief summary. We also thank them for the appreciation they have shown.

**Reviewer #2:** This is a comprehensive study of a series of Toxoplasma gondii prolyl-tRNA synthetase (PRS) inhibitors based on the 1-(pyridine-4-yl) pyrrolidin-2-one (PPL) scaffold that targets the ATP binding pocket of the PRS enzyme. The work is thorough and well thought out, clearly demonstrating these inhibitors are targeting the Tg PRS (through generation of resistant mutants) and comparison of the binding of these inhibitors to human vs parasite PRS by thermal shift binding assays and crystallization studies. The work is important and adds considerable information to further the development of PRS inhibitors for anti-apicomplexan drug development. However this reviewer has some concerns with the narrowness of the selectivity indices, the lack of CC50 data on the host cells the parasites are grown in, and the validity of EC50 values that are close to or exceed the CC50 values in the case of resistant mutants. Overall, the manuscript could also use some careful grammatical editing, and the authors should confirm that statements made in the text match the data shown in the figures.

**Reply:** We agree with the reviewer that based on CC50 determined from ARPE19 and MDA231 mammalian cells, the selectivity indices appear quite low, which is unfortunate from a clinical perspective but this study does not claim discovery of candidate drugs. We chose to use these cell lines rather than the HFF because they are quite relevant for testing drug biological activity as they are more sensitive to drug treatment than HFF, which are quite robust and would overestimate the selectivity indices.

**Reviewer #3:** In this manuscript, the authors provide evidence that five ATP-mimetic compounds developed to bind human and Plasmodium prolyl-tRNA synthetase (PRS) also bind PRS from Toxoplasma gondii (Tg) and inhibit its enzymatic activity and parasitic growth. The authors show these compounds inhibit Tg growth in HFFs and have approximately 1-10-fold selectivity by cytotoxicity in an epithelial and breast cell line and up to a 14x lower EC50 than the standard of care Tg drug pyrimethamine. They used a chemical mutagenesis screen to select for parasites resistant to the compound with the lowest EC50, L35, and discovered two mutations of interest in the PRS coding region, T477A and T592S. They confirmed at least one of these mutations confer resistance of the parasite to all five compounds, though which mutants are resistant to which compounds varied. The authors then proceed to characterize the biochemical inhibition of both the Tg and human PRS enzymes with each of these compounds and found that 3 are substantially more selective for the Tg PRS than human. The enzymatic data were confirmed with thermal shift data in the presence and absence of PRS substrates. Finally, the authors supplied excellent high resolution x-ray crystal structures of all five inhibitors bound to Tg PRS and three bound to human PRS. These structures revealed a consistent core binding mode of the PPL scaffold with specific nuanced differences in binding between the different compounds which provide structural evidence for differences in selectivity and activity. The authors conclude that these compounds are potential anti-infectives for Tg disease and the structural and biochemical data form a solid backbone for structure-guided development of these compounds as novel Tg drugs.
**Reply:** We greatly appreciate and thank the reviewer for their feedback.

**Impressions:**

This paper provides a strong argument for these PPL compounds to be further developed as anti-infectives for Tg. The compounds already show selectivity over human PRS and reasonable biological selectivity with cell lines. The emergence of biological mutants with resistance is always a concern with any new drug development; however, the authors have identified two critical mutants at this early stage which provides important testing points for new derivatives. A weakness is the lack of biochemical and structural evaluations of these or other mutants, which would be useful to cross-validate the study conclusions here.

The crystal structures are a major strength of this manuscript. All structures are of high resolution with all but one greater than 2.5 A and 6/14 less than 2 A. Model fitting statistics suggest good to excellent agreement in the model to the experimental data and ligand fit was performed diligently. Minor issues are present with data presentation and numbering/unit clarity and consistency, and these are detailed below. All but one structure was available in the PDB for review and at least one was previously deposited and included in a different publication by the same group. The crystallographic data was included for easy comparison and appreciated.

The authors provide compelling data to explain selectivity and activity differences. The discussion succinctly summarizes these findings, though could be expanded to discuss other resistance mechanisms, such as relevance of the adaptive proline response mentioned in the introduction, and whether novel derivatives would need to avoid the proline binding site to avoid these mechanisms.

Overall, this paper represents an important step in identifying new compounds to develop into therapies for Tg infections, which is a major unmet need in infectious disease pharmacology. The structural, biochemical, and biological data presented here will not only be useful in further development but may have applications for other parasites, such as Plasmodium, and may become a new class of therapeutic inhibitors.

**Reply:** We thank you all for the attention to detail and appreciation you have exhibited in reviewing our work. This is highly encouraging and motivating.

**Part II – Major Issues: Key Experiments Required for Acceptance**

Please use this section to detail the key new experiments or modifications of existing experiments that should be absolutely required to validate study conclusions.

Generally, there should be no more than 3 such required experiments or major modifications for a "Major Revision" recommendation. If more than 3 experiments are necessary to validate the study conclusions, then you are encouraged to recommend "Reject".

**Reviewer #1:**

This reviewer is unable to comment on the structural analysis, since it is far outside of his expertise. All other experiments have been performed to the highest standard and there are no major concerns.

**Reply:** We thank the reviewer for their feedback and greatly appreciate the support.

**Reviewer #2:**

**Query:** Introduction/Fig 1: It is stated that these inhibitors were developed to target HsPRS as anti-fibrosis drugs. Were these modified in any way to optimize the interaction with TgPRS or are these the inhibitors developed to target HsPRS, and were identified by screening for activity against Tg or TgPRS? The author also mention that PfPRS was also targeted with PPL derivatives. Are these the derivatives described here as having activity against TgPRS? Please clarify.

**Reply:** These drugs have not been modified in any way to optimize binding with either *Tg* or *Tg*PRS. These were identified by screening for activity against *Tg*, and subsequently were tested for enzymatic inhibition. Yes, these exact PPL derivatives were tested against *Pf*PRS as well (manuscript in preparation). This has been mentioned in the introduction for clarity of the readers on Pgs 3-4, lines 61-70. Please see below.

“In 2017, Takeda Pharmaceuticals disclosed a new class of *Hs*PRS inhibitors to treat fibrosis.^34^ Unlike HFG and analogs, which span the A76 and proline-binding sites and thus interact in an ATP-uncompetitive manner, these new 1-(pyridin-4-yl) pyrrolidin-2-one (PPL) derivatives target the ATP-binding pocket adjacent to the active site. Some of these compounds, e.g., T-3767758 displayed proline-uncompetitive steady-state kinetics for *Hs*PRS.^30^ Based on this class of inhibitors, Okaniwa et al. identified PPL derivatives as a new class of antimalarials that bind to the ATP-site with double-digit nanomolar activity against *Plasmodium* *falciparum* (*Pf*) and *Plasmodium* *vivax* (*Pv*) strains.^32^“

**Query:** Fig 2 C and D-the selectivity indices overall are very narrow for these PRS inhibitors. Because of this, it would be best if the CC50 and SI for the HFF host cells was also provided. It is not sufficient to just visually examine the host cells (line 228). **Reply:** We agree with the reviewer that based on CC50 determined from ARPE19 and MDA231 mammalian cells, the selectivity indices appear quite low. However, we chose to use these cell lines rather than the HFF because they are relevant to test drug biological activity as they are more sensitive to drug treatment than HFF, which are quite robust and would overestimate the selectivity indices.

**Query:** In Fig 2: L36 has an EC50 of 5800 nM (Fig 2A). The CC50 for this compound was never evaluated (Fig 2C and D; and Fig 4E). Is the compound specific for the parasite or is it killing the parasite via effects on the host cell?

**Reply:** We confirm that the CC50s for L36 were evaluated. Note that the notation "n.d.; not detected" in the figures indicates that a CC50 value could not be evaluated because no inhibition could be observed even at the maximum concentration tested (> 33000 nM). Therefore, no SI could be calculated.

**Query:** Lines 255-256: “Notably, the mutation T477A significantly impaired the growth of L35-treated parasites (Fig. 3D lower panel, S2C) compared to pyrimethamine-treated parasites” It is unclear what comparison is being made here, or what point the authors are trying to make.

**Reply:** This line here has been edited to clearly elucidate that the plaque size of the T592S mutant was somewhat higher than that of the T477A mutant. The revised text reads, “Notably, the plaque size of the T592S mutant was somewhat higher than that of the T477A mutant, however upon treatment with L35 – the trend was reversed (although the results were non-significant). Furthermore, we would like to point out that mutant T477A was ~ 3X more resistant towards L35 (186 nM for T477A vs 76 nM for T592S) than the T592S mutant. However, both these mutants (T477A and T592S) are equivalently susceptible to pyrimethamine as wild-type parasites (as is expected by virtue of mutual exclusion of mechanisms of action).“ These changes have been done in lines 271-277, page 11.

**Query:** Fig 4E. It is unclear how the EC50s were determined as, in some cases, the EC50 exceeds the CC50 or is very close to the CC50 (For example L95 against T592S and L97 against T477A). Surely inhibition of host cells would affect growth of the parasite and confound calculation of the EC50?

**Reply:** A paragraph describing the CC50 determination procedure for the mammalian cells has been added to the *Methodology* section. It should be noted that EC50s were determined using a monolayer of confluent HFF host cells with reduced metabolic activity as opposed to dividing cells – they are presumably less sensitive to drugs than exponentially proliferating tachyzoites. We agree with the reviewer that at these high drug concentrations host cells could be affected, resulting in an underestimation of the EC50 for parasites. However, this does not compromise our interpretation of the data regarding the drug resistance of *Tg*PRS mutant parasites and the low selectivity indices observed with these compounds (<100).

**Query:** Also in Fig 4E, the T592S mutation made the parasite considerably more susceptible to derivative L36. Perhaps the authors could comment on this?

**Reply:** Yes, this trend could be addressed as possibly due to the enantiopreference between L35 and L36 – which in itself is due to specific interactions which are reversed based on this interaction based on this single amino acid change. This has now been mentioned on Pg. 12, lines 309-310 for clarity of the readers.

**Reviewer #3:** This study identified two PRS mutants with reduced biological susceptibility to L35. Biological resistance of these mutants was validated by complimenting wt Tg with the same mutants which confirmed the phenotype. The mechanism by which these mutants reduce susceptibility was not validated. An evaluation of the biochemical activity of these compounds with purified PRS T477A and PRS T592S would be useful to suggest whether these mutations reduce biological L35 activity due to lower enzymatic inhibition/binding or via some other indirect mechanism.

The co-crystal structures are of high resolution and the Fo-Fc difference maps suggest accurate placement of the ligands into the electron density, though the lowest resolution structure (7FAL) has less connectivity for assignment. It would be useful to have a biochemical validation of these inhibitor models by mutating a residue that the model shows interacts with the inhibitors (such as but not limited to T482) and observing any reduced binding affinity/activity biochemically. Understandably any active site mutagenesis may impact enzymatic activity, so if selecting a mutant that does not impact conformation/activity is not possible, computational predictions such as docking in a predicted mutant model may be a useful surrogate test to validate the placement of ligands in the model.

**Reply:** We thank the reviewer for their comments. Indeed, the inhibitory activity of the compounds on the recombinant *Tg*PRS T477A and *Tg*PRS T592S enzymes was not evaluated. While it is true that for some inhibitory compounds, mutagenesis approaches can detect resistance genes that either (i) alter the bioavailability of certain compounds (e.g. mutations of the efflux channel in P. falciparum that alter the uptake of the compound), (ii) degrade or chemically alter certain compounds, (iii) act allosterically on the function of the target protein, and/or (iv) act by lowering the affinity of the target protein for the inhibitory compound. In the current manuscript, several converging reasons point to a mechanism of resistance based on the latter example – i.e. lowering the affinity to the target protein. Firstly, L35 is a derivative of a known ATP-mimetic scaffold that targets the ATP-binding pocket, and secondly, our forward genetic approach identified mutations within the *Tg*PRS gene conferring resistance to L35, thus ruling out resistance mechanisms based on efflux pumps.

**Part III – Minor Issues: Editorial and Data Presentation Modifications**

Please use this section for editorial suggestions as well as relatively minor modifications of existing data that would enhance clarity.

**Reviewer #1:**

**Query:** 1.) line 255ff: “Notably, the mutation T477A significantly impaired the growth of L35-treated parasites (Fig. 3D lower panel, S2C) compared to pyrimethamine-treated parasites (Fig. 3D upper panel, S2C).”

T477A confers more resistance compared to T592S? In contrast (and as expected) treatment with pyrimethamine shows similar effect for WT and mutant parasites. Therefore, this sentence should read:

“Notably, the parasites with the T477A mutation significantly overcame inhibition of growth in presence of L35 (Fig. 3D lower panel, S2C) compared to pyrimethamine-treatment (Fig. 3D upper panel, S2C).

**Reply:** We thank the reviewers for this suggestion. Yes, this rephrasing does make the message clearer. And we have rephrased the same and added a point about the fitness of these mutants using plaque size as a proxy on lines 255-262.

**Query:** Figure 2F not described in the text.

**Reply:** We thank the reviewer for pointing this out and have now added its mention in the text at Line 245.

**Query:** Figure 4F is not described in the legend

**Reply:** We thank the reviewer for this comment. We have added the same now.

**Reviewer #2: Minor comments:**

**Query:** Figs 3D and Fig 4A-D would be better presented as tables, especially as the scales on each graph vary.

**Reply:** We have addressed the reviewer’s concern and added tables as supplementary data in addition to the figures. We would like to keep the figures as we feel that the graphical representation of data is more visually engaging than tables.

**Table S5 Summary of EC50 values of WT and mutants against L35.**

| **Mutant Parasite** | **EC_50_ Treated-PYR (nM)** | **EC_50_ Treated-L95 (nM)** | **EC_50_ Treated-L96 (nM)** | **EC_50_ Treated-L97 (nM)** | **EC_50_ Treated-L35 (nM)** | **EC_50_ Treated-L36 (nM)** |
| --- | --- | --- | --- | --- | --- | --- |
| WT | 174 ± 60 | 277 ± 6 | 106 ± 36 | 544 ± 130 | 31 ± 12 | 5800 ± 350 |
| T477A | 198 ± 70 | 174 ± 120 | 249 ± 110 | 1800 ± 410 | 186 ± 12 | 7800 ± 700 |
| T592S | 241 ± 140 | 1000 ± 170 | 525 ± 7 | 470 ± 28 | 76 ± 19 | 1600 ± 310 |

**Query:** Fig 3 C vs Fig 3D. the green and orange colors which represent the mutants are reversed between the figures (T477A is orange in C and green in D).

**Reply:** We thank the reviewers for this feedback. We have corrected this in the figure accordingly.

**Query:** Fig 4F lacks a legend.

**Reply:** We thank the reviewer for this comment. We have added the same now. “The catalytic cavity of *Tg*PRS is shown with two inhibitors along with their electron densities – HFG (green) and L95 (red) – bound to the tRNA and L-pro binding sites and the ATP binding site respectively. The ribbon of PRS is in cyan whilst the resistance-conferring mutating residues T477 and T592 to L35 are shown in purple.”

**Query:** Fig 5 A and B have no key to indicate which inhibitor is represented by which curve. Perhaps these figures would be better in the supplemental as the data is summarized in Fig 5C.

**Reply:** We thank the reviewer for this feedback. The corresponding key is shown alongside the new figure now.

**Query:** Fig 5 D and E: Thermal shift data shown in Fig 5D and E are described in lines 295-298, but the numbers in the text do not match the numbers in the table in Fig 5E

**Reply:** We thank the reviewer for this concern. We would like to clarify that the lines 295-298 describe the enzyme inhibition data and the enzymatic selectivity indices as shown in Figs 5A, 5B and 5C. The TSA data from Fig 5D and 5E is described in lines 305-308. Furthermore, the numbers discussed are the delta Tm values only and not the Tm values themselves.

**Reviewer #3:**

**Query:** Line 27- abstract- unclear from sentence for which organism PRS is a validated drug target

**Reply:** For clarity, we have edited the text in the revised manuscript to read – “The prolyl-tRNA synthetase (PRS) is a validated drug target for febrifugine and its synthetic analog halofuginone (HFG) against multiple apicomplexan parasites like *Plasmodium falciparum* and *Toxoplasma gondii*. Here, a novel ATP-mimetic centered on 1-(pyridin-4-yl) pyrrolidin-2-one (PPL) scaffold has been validated to bind *Toxoplasma gondii* PRS and kill toxoplasma parasites.” Pg. 2, lines 28-29.

**Query:** Line 131- sequence length of Tg PRS is indicated but not human PRS…appears to be approx. 1015-1506 from deposited structures

**Reply:** We thank the reviewer for this feedback. This point has been added at line 131. The text now reads, “PRSs from *Tg*PRS: length 334 – 830 (TGME49_219850 S8G8I1) and *Hs*PRS: length 1015 – 1506 (EPRS (EARS, PARS, PIG32) Q3KQZ8_HUMAN) were purchased as a gBlock (Integrated DNA Technologies, Leuven, Belgium). “

**Query:** Line 181- “concentrations greater than their Km” is a bit vague…consider indicating actual concentration used or scale factor from Km

**Reply:** We thank the reviewer for this feedback. This point has been addressed as 1.5X concentrations of their K_m_ values at line 182.

**Query:** Line 184-193- Please indicate the concentration of the protein for crystallization somewhere in the methods (or table S2 if all are different)

**Reply:** We thank the reviewer for pointing this out. These have been added at line 185. For *Hs*PRS – 12-15 mg mL^-1^ and for *Tg*PRS – 10-14mg mL^-1^.

**Query:** Line 190- were the drugs dissolved in DMSO before being added to the protein? If so, what is the final DMSO concentration or range?

**Reply:** Yes, the drugs were dissolved in 100% DMSO before being added to the protein. The final concentration of DMSO whilst crystallizing is 2.5 – 7.5 %.

**Query:** Line 234 & Fig. 2A- values given in text are nanomolar and in figure are micromolar…readability would be improved with consistent units.

**Reply:** We thank the reviewer for pointing this out. This has been made consistent throughout the manuscript now.

**Query:** Line 326 & 328-results- Figs. 7 & 8 were called out before Fig. 6…consider reordering/renaming

**Reply:** We thank the reviewer for their feedback. We have considered their point and removed the reference to figures 7 and 8 on Pg.14, line 369, prior to the mention of Figure 6.

**Query:** Line 327 & Line 661 Fig. 6 caption- Text (327) indicates inhibitors were verified at 3 sigma, but the figure (661) shows OMIT contour maps at sigma. Please confirm whether OMIT maps are contoured at 1 sigma or 3 sigma.

**Reply:** The OMIT maps are contoured at 3 σ. And this has been clarified in line 675.

**Query:** Line 357 & Fig. S3- Interactions and H-bonds are labeled in panels A & B but not in C-E.

**Reply:** We thank the reviewer for their comment. These figures have been sorted out now to show the bonds.

**Query:** Additionally, the text states that there is no H-bond with L36 and R481, but the distance between the guanidinium and the methylpyrazole of L35 (7FAN) is approx. 3.4A and the distance between the guanidinium and the methylpyrazole of L36 (7FAL) is approx. 3.3A…what criteria was used to determine H-bond existence? It could be helpful to show H-bond distances in Fig. S3 if space allows/visually possible.

**Reply:** The criterion used for the determination of H-bond existence was done with the help of the PLIP server – which considers the definition to be 2.7-3.3A, not including the limits. And wherever there were multiple ligand-protein molecules within a structure – the presence of an H-bond was confirmed only in the presence of it in all molecules.

**Query:** Line 371 & Fig. 8B- “motif 1” indicated in text but labeled as “motif” in Fig. 8 panel B.

**Reply:** This text has been edited in the revised version of the manuscript to read, “Significant structural variations within the catalytic domain (1016-1296): ATP pocket (1152-1165), loop (1210-1220), and motif (1084-1115) were observed for the three co-crystallized structures (Fig. 8 B).” on Pg. 16, lines 412-414.

**Query:** Line 379 & 380- please indicate which species’ residues are in parenthesis and which are not

**Reply:** The style of denoting residue and number has been changed – such that the first part before the slash is the *Hs* corresponding data and that after the slash corresponds to the *Tg* one.

**Query:** Fig. 5A&B- which color/shape is which compound? Same as in panel D?

**Reply:** We thank the reviewer for this feedback. The corresponding key is shown alongside the new figure now.

Fig. 5C- Units in text are nM but in figure are µM.

**Reply:** We thank the reviewer for pointing this inconsistency out. The units in all the figures, including 5C have been changed to nM for consistency.

Supplemental Table S3-

**Query:** The legend calls out Favored/Allowed/Outlier for the Ramachandran plot but only two numbers are shown in the boxes ex: 98/2. They appear to be Favored/Allowed only…please clarify the callout in the legend or add a decimal to indicate all 3, such as 97.5/2.1/0.4 since there is a small percentage (<0.5%) of outliers, and some (such as F479) are close to the inhibitor binding site and might be of interest to note if inhibitors influence rotamers.

**Reply:** We thank the reviewer for this comment. The f/a/o break-up has been denoted as defined now.

**Supplementary Table S3.** Summary PDB entries associated with this manuscript.

| **Name** | **PDB ID** | **SG** | **Res. (Å)** | **R_w_/R_f_ (%)** | **B^@^ (Å^2^)** | **CS^** | **Rama* (%)** |
| --- | --- | --- | --- | --- | --- | --- | --- |
| *Tg*PRS-L95 | 7EVV | C2 | 1.70 | 15.3/18.1 | 32 | 3 | 98.5/1.3/0.2 |
| *Tg*PRS-L95 | 7VC1 | P2_1_ | 1.89 | 17.4/21.1 | 40 | 3 | 98.4/1.5/0.1 |
| *Tg*PRS-L96 | 7FAK | C2 | 1.90 | 17.6/20.5 | 43 | 6 | 97.9/2.1/0.0 |
| *Tg*PRS-L96 | 7VC2 | P2_1_ | 2.10 | 17.7/21.8 | 51 | 5 | 98.1/1.7/0.2 |
| *Tg*PRS-L97 | 7FAM | C2 | 2.42 | 18.2/23.3 | 56 | 5 | 97.3/2.5/0.2 |
| *Tg*PRS-L97 | 7VC3 | C2 | 1.97 | 17.5/21.7 | 54 | 3 | 98.1/1.7/0.2 |
| *Tg*PRS-T35 | 7FAN | C2 | 1.77 | 17.5/21.8 | 42 | 4 | 97.7/2.1/0.2 |
| *Tg*PRS-T36 | 7FAL | P2_1_ | 3.22 | 22.9/25.3 | 53 | 8 | 96.4/3.2/0.4 |
| *Hs*PRS-L95 | 7F9B | P2_1_ | 2.0 | 18.9/23.6 | 47 | 4 | 98.5/1.3/0.2 |
| *Hs*PRS-L95 | 7F98 | P2_1_2_1_2_1_ | 2.0 | 19.0/23.5 | 53 | 5 | 98.4/1.4/0.2 |
| *Hs*PRS-L96 | 7F9C | P2_1_ | 2.20 | 18.8/25.3 | 57 | 5 | 97.7/2.1/0.2 |
| *Hs*PRS-L96 | 7F99 | P2_1_ | 1.99 | 18.1/22.8 | 43 | 3 | 98.4/1.4/0.2 |
| *Hs*PRS-L96 | 7F9D | P2_1_2_1_2_1_ | 2.5 | 18.7/26.3 | 54 | 5 | 98.7/1.0/0.3 |
| *Hs*PRS-L97 | 7F9A | P2_1_2_1_2_1_ | 2.0 | 19.5/24.2 | 61 | 4 | 98.4/1.3/0.3 |

SG - Space group, Res. - Resolution, Fo,Fc Cor - Fo,Fc correlation, B@ (Å^2^) - Average B for all atoms, CS^ -Clashscore, Rama* (%) - Ramachandran plot Favoured/Allowed/Outliers

**Query:** The table could benefit from including additional information typical to the standard tables for reporting crystallographic data, and similar to Table 1 in the authors’ companion publication (Manickam, Y. et. al. 2022). Although adding all of the collection and refinement statistics in the table may be cumbersome, reporting the resolution as shells and highest shell would be useful, as well as including I/sigmaI, completeness, and RMS deviations.

**Reply:** We thank the reviewer for this suggestion and understanding that adding these may be cumbersome. However, we have added the shells, I/sigma(I), completeness and RMSD data along with any other relevant details.

**Query:** All structures were available in the PDB for review except 7FAK was still on hold. Although it was not required for this review, as the structures presented here were all of excellent quality, and this specific inhibitor had a complimentary structure in a different space group, the authors should be aware of the hold in case there was a mistake in the release date. If not, no concerns.

**Reply:** The PDB 7FAK is not on hold anymore and will be public soon.

**Supplementary Table S4.** Summary of data collection and refinement statistics. Statistics for the highest-resolution shell are shown in parentheses.

| **PRS-Inhibitor** | **TgPRS-L95^!^** | **TgPRS-L95** | **TgPRS-L96** |
| --- | --- | --- | --- |
| PDB code | 7EVV | 7VC1 | 7FAK |
| **Data collection and processing** |  |  |  |
| Diffraction source | PROXIMA-1 (PX1) | PROXIMA-1 (PX1) | DLS I04 |
| Wavelength (Å) | 0.979 | 0.9786 | 0.9795 |
| Detector | EIGER X 16M | EIGER X 16M |  |
| Total rotation range (°) | 360 | 360 | 720 |
| Space group | C2 | P2_1_ | C2 |
| a, b, c (Å) | 111.35, 75.20, 74.74 | 76.89, 81.80, 104.66 | 113.62, 74.23, 74.65 |
| α, β, γ (°) | 90.0, 110.7, 90.0 | 90.0, 102.6, 90.0 | 90.0, 111.1, 90.0 |
| Resolution (Å) | 41.85–1.70 (1.71–1.70) | 47.29–1.89 (2.01–1.89) | 60.81–1.89 (1.94–1.89) |
| No. of unique reflections | 62494 (9672) | 100749 (15940) | 45141 (2157) |
| Completeness (%) | 99.1 (95.7) | 99.6 (97.9) | 98.6 (94.9) |
| Redundancy | 2.2 (2.3) | 7.0 (6.7) | 6.6 (5.7) |
| I/σI | 13.3 (2.9) | 10.1 (1.5) | 8.7 (1.3) |
| Half-set correlation CC_1/2_ ^*^ | 1.0 (0.8) | 1.0 (0.8) | 1.0 (0.5) |
| R_meas_^**^ | 0.089 (6.02) | 0.118 (1.163) | 0.125 (0.890) |
| Wilson B-factor (Å^2^) | 26.2 | 33.0 | 26.2 |
| **Structure refinement** |  |  |  |
| Resolution (Å) | 31.52–1.70 | 47.29–1.89 | 60.84–1.89 |
| No. of reﬂections /test set | 62474 / 3122 | 100697 / 5035 | 41502 / 2199 |
| R_work_/ R_free_ (%) | 15.3 / 18.1 | 17.4 / 21.1 | 18.1 / 21.6 |
| No. of molecules in ASU | 1 | 2 | 1 |
| No. of protein residues | 490 | 962 | 483 |
| No. of protein atoms/water molecules | 4040 / 287 | 7794 / 628 | 3928 / 286 |
| No. of ligand atoms | 36 + 8 | 36 + 8 | 37 + 8 |
| No. of ions^#^ | 1 | 4 | - |
| No. of solvent atoms^$^ | 16 | 52 | 52 |
| **R.m.s deviations** |  |  |  |
| Bond lengths (Å) | 0.018 | 0.007 | 0.016 |
| Bond angles (º) | 1.508 | 0.895 | 1.309 |
| **Average B factors (Å^2^)** |  |  |  |
| Protein / water | 31.6 / 37.9 | 39.8 / 48.1 | 30.8 / 37.82 |
| Ligand atoms / ions | 21.7 / 77.4 | 27.4 / 69.9 | 35.33 / - |
| **Ramachandran plot** |  |  |  |
| Favoured / Allowed / Outliers (%) | 98.5 / 1.3 / 0.2 | 98.4 / 1.5 / 0.1 | 97.9 / 2.1 / 0.0 |

| **PRS-Inhibitor** | **TgPRS-L96** | **TgPRS-L97** | **TgPRS-L97** |
| --- | --- | --- | --- |
| PDB code | 7VC2 | 7FAM | 7VC3 |
| **Data collection and processing** |  |  |  |
| Diffraction source | PROXIMA 2 | PROXIMA 2 | DLS I24 |
| Wavelength (Å) | 0.9804 | 0.9804 | 0.9686 |
| Detector | EIGER X 9M | EIGER X 9M |  |
| Total rotation range (°) | 360 | 360 | 360 |
| Space group | P2_1_ | C2 | C2 |
| a, b, c (Å) | 77.10, 81.62, 104.88 | 115.49, 74.04, 74.85 | 114.73, 73.64, 74.91 |
| α, β, γ (°) | 90.0, 102.68, 90.0 | 90.0, 111.24, 90.0 | 90.0, 111.38, 90.0 |
| Resolution (Å) | 47.42–2.10 (2.23–2.10) | 52.87–2.42 (2.48–2.42) | 60.64–1.97 (2.01–1.97) |
| No. of unique reflections | 74227 (11607) | 22556 (3400) | 40410 (2008) |
| Completeness (%) | 99.4 (96.9) | 100 (91.0) | 98.6 (97.9) |
| Redundancy | 6.9 | 6.8 | 6.9 (7.1) |
| I/σI | 14.3 (1.7) | 7.4 (1.4) | 10.7 (0.6) |
| Half-set correlation CC_1/2_ | 1.0 (0.7) | 1.0 (0.9) | 1.0 (0.4) |
| R_meas_ | 0.091 (0.977) | 0.092 (0.993) | 0.109 (4.191) |
| Wilson B-factor (Å^2^) | 43.2 | 48.7 | 44.2 |
| **Structure refinement** |  |  |  |
| Resolution (Å) | 47.42–2.10 | 53.87–2.42 | 52.68–1.97 |
| No. of reﬂections /test set | 145446 / 7280 | 22556 / 1076 | 39532 / 1981 |
| R_work_/ R_free_ (%) | 17.7 / 21.8 | 18.2 / 23.3 | 17.5 / 21.7 |
| No. of molecules in ASU | 2 | 1 | 1 |
| No. of protein residues | 947 | 480 | 483 |
| No. of protein atoms/water molecules | 7654 / 416 | 3916 / 47 | 3911 / 130 |
| No. of ligand atoms | 90 | 43 | 43 |
| No. of ions^#^ | 1 | - | 12 |
| No. of solvent atoms^$^ | 48 | - | 26 |
| **R.m.s deviations** |  |  |  |
| Bond lengths (Å) | 0.008 | 0.009 | 0.008 |
| Bond angles (º) | 0.964 | 0.972 | 0.928 |
| **Average B factors (Å^2^)** |  |  |  |
| Protein / water | 51.0 / 54.1 | 57.6 / 51.2 | 53.6 / 55.1 |
| Ligand atoms / ions | 54.5 / 85.4 | 49.9 / - | 42.7 / 91.4 |
| **Ramachandran plot** |  |  |  |
| Favoured / Allowed / Outliers (%) | 98.1 / 1.7 / 0.2 | 97.3 / 2.5 / 0.2 | 98.1 / 1.7 / 0.2 |

| **PRS-Inhibitor** | **TgPRS-L35** | **TgPRS-L36** | **HsPRS-L95** |
| --- | --- | --- | --- |
| PDB code | 7FAN | 7FAL | 7F9B |
| **Data collection and processing** |  |  |  |
| Diffraction source | PROXIMA-1 (PX1) | DLS I04 | PROXIMA 2 |
| Wavelength (Å) | 0.9786 | 0.9795 | 0.9799 |
| Detector | EIGER X 16M |  | EIGER X 9M |
| Total rotation range (°) | 360 | 720 | 360 |
| Space group | C2 | P2_1_ | P2_1_ |
| a, b, c (Å) | 114.52, 74.30, 75.07 | 76.01. 86.33, 104.46 | 70.73, 90.96, 83.49 |
| α, β, γ (°) | 90.0, 111.2, 90.0 | 90.0, 102.33, 90.0 | 90.0, 110.03, 90.0 |
| Resolution (Å) | 52,65–1.77 (1.82–1.77) | 67.25–3.22 (3.33–3.22) | 45.48–2.0 (2.11–2.0) |
| No. of unique reflections | 57267 (4208) | 22703 (1124) | 66905 (10359) |
| Completeness (%) | 100 (100) | 99.9 (100) | 98.4 (94.7) |
| Redundancy | 6.5 (5.9) | 6.9 (6.9) | 6.9 |
| I/σI | 9.2 (1.2) | 4.8 (1.1) | 9.9 (1.1) |
| Half-set correlation CC_1/2_ (%) | 1.0 (0.5) | 1.0 (0.5) | 1.0 (0.4) |
| R_meas_ | 0.091 (1.301) | 0.480 (2.230) | 0.146 (1.553) |
| Wilson B-factor (Å^2^) | 30.1 | 50.4 | 40.0 |
| **Structure refinement** |  |  |  |
| Resolution (Å) | 36.53–1.77 | 54.74–3.22 | 45.48–2.0 |
| No. of reﬂections /test set | 57207 / 2863 | 21485 / 1009 | 66490 / 3328 |
| R_work_/ R_free_ (%) | 17.5 / 21.8 | 23.3 / 25.4 | 18.9 / 23.6 |
| No. of molecules in ASU | 1 | 2 | 2 |
| No. of protein residues | 486 | 958 | 968 |
| No. of protein atoms/water molecules | 3936 / 209 | 7816 / 7 | 7597 / 266 |
| No. of ligand atoms | 44 | 88 | 72 |
| No. of ions^#^ | - | - | 11 |
| No. of solvent atoms^$^ | - | - | - |
| **R.m.s deviations** |  |  |  |
| Bond lengths (Å) | 0.017 | 0.005 | 0.008 |
| Bond angles (º) | 1.444 | 0.967 | 0.965 |
| **Average B factors (Å^2^)** |  |  |  |
| Protein / water | 41.6 / 45.2 | 52.7 / 21.1 | 46.9 / 48.3 |
| Ligand atoms / ions | 31.5 / - | 56.4 / - | 35.5 / 66.1 |
| **Ramachandran plot** |  |  |  |
| Favoured / Allowed / Outliers (%) | 97.7 / 2.1 / 0.2 | 96.4 / 3.2 / 0.4 | 98.5 / 1.3 / 0.2 |

| **PRS-Inhibitor** | **HsPRS-L95** | **HsPRS-L96** | **HsPRS-L96** |
| --- | --- | --- | --- |
| PDB code | 7F98 | 7F9C | 7F99 |
| **Data collection and processing** |  |  |  |
| Diffraction source | PROXIMA 2 | PROXIMA 2 | PROXIMA 2 |
| Wavelength (Å) | 0.9801 | 0.9801 | 0.9801 |
| Detector | EIGER X 9M | EIGER X 9M | EIGER X 9M |
| Total rotation range (°) | 360 | 360 | 360 |
| Space group | P2_1_2_1_2_1_ | P2_1_ | P2_1_ |
| a, b, c (Å) | 70.72, 196.11, 145.29 | 70.72, 196.11, 145.29 | 70.47, 91.52, 83.34 |
| α, β, γ (°) | 90.0, 90.0, 90.0 | 90.0, 90.0, 90.0 | 90.0, 110.11, 90.0 |
| Resolution (Å) | 59.94–2.0 (2.12–2.0) | 62.29–2.20 (2.33–2.20) | 78.25–1.98 (2.01–1.98) |
| No. of unique reflections | 74529 (11755) | 50785 (8075) | 69229 (3404) |
| Completeness (%) | 99.7 (98.4) | 99.6 (98.8) | 99.9 (98.4) |
| Redundancy | 13.4 | 6.2 | 6.7 |
| I/σI | 12.6 (0.8) | 7.3 (0.5) | 9.5 (2.0) |
| Half-set correlation CC_1/2_ | 1.0 (0.5) | 1.0 (0.4) | 1.0 (0.5) |
| R_meas_ | 0.13(2.65) | 0.18 (2.22) | 0.12 (0.854) |
| Wilson B-factor (Å^2^) | 46.8 | 55.3 | 31.7 |
| **Structure refinement** |  |  |  |
| Resolution (Å) | 59.94–2.0 | 62.29–2.20 | 78.25–1.98 |
| No. of reﬂections /test set | 74444 / 3722 | 99589 / 4976^&^ | 69187 / 3366 |
| R_work_/ R_free_ (%) | 19.0 / 23.5 | 18.8 / 25.3 | 18.1 / 22.8 |
| No. of molecules in ASU | 2 | 2 | 2 |
| No. of protein residues | 983 | 974 | 972 |
| No. of protein atoms/water molecules | 7801 / 296 | 7709 / 173 | 7011 / 407 |
| No. of ligand atoms | 72 | 90 | 90 |
| No. of ions^#^ | 12 | 13 | 9 |
| No. of solvent atoms^$^ | - | - | - |
| **R.m.s deviations** |  |  |  |
| Bond lengths (Å) | 0.008 | 0.008 | 0.007 |
| Bond angles (º) | 0.958 | 0.998 | 0.929 |
| **Average B factors (Å^2^)** |  |  |  |
| Protein / water | 53.9 / 53.6 | 57.2 / 52.65 | 42.9 / 44.8 |
| Ligand atoms / ions | 41.9 / 83.6 | 48.6 / 70.3 | 27.2 / 62.7 |
| **Ramachandran plot** |  |  |  |
| Favoured / Allowed / Outliers (%) | 98.4 / 1.4 / 0.2 | 97.7 / 2.1 / 0.2 | 98.4 / 1.4 / 0.2 |

| **PRS-Inhibitor** | **HsPRS-L96** | **HsPRS-L97** |
| --- | --- | --- |
| PDB code | 7F9D | 7F9A |
| **Data collection and processing** |  |  |
| Diffraction source | PROXIMA 2 | PROXIMA 2 |
| Wavelength (Å) | 0.980 | 0.9801 |
| Detector | EIGER X 9M | EIGER X 9M |
| Total rotation range (°) | 360 | 360 |
| Space group | P2_1_2_1_2_1_ | P2_1_ |
| a, b, c (Å) | 70.49, 106.13, 145.55 | 70.72, 196.11, 145.29 |
| α, β, γ (°) | 90.0, 90.0, 90.0 | 90.0, 90.0, 90.0 |
| Resolution (Å) | 44.13-2.50 (2.65-2.50) | 59.93-2.0 (2.12-2.0) |
| No. of unique reflections | 38588 (6065) | 74577 (11817) |
| Completeness (%) | 99.7 (98.5) | 99.8 (98.9) |
| Redundancy | 13.2 | 15.0 |
| I/σI | 9.2 (1.5) | 12.5 (0.5) |
| Half-set correlation CC_1/2_ | 1.0 (0.6) | 1.0 (0.5) |
| R_meas_ | 0.24 (1.50) | 0.13 (2.61) |
| Wilson B-factor (Å^2^) | 52.4 | 53.3 |
| **Structure refinement** |  |  |
| Resolution (Å) | 44.13-2.50 | 59.93-2.0 |
| No. of reﬂections /test set | 73020 / 3650^&^ | 74344 / 3721 |
| R_work_/ R_free_ (%) | 18.7 / 26.3 | 19.5 / 24.2 |
| No. of molecules in ASU | 2 | 2 |
| No. of protein residues | 980 | 987 |
| No. of protein atoms/water molecules | 7767 / 113 | 7787 / 236 |
| No. of ligand atoms | 90 | 86 |
| No. of ions^#^ | 12 | 15 |
| No. of solvent atoms^$^ | - | - |
| **R.m.s deviations** |  |  |
| Bond lengths (Å) | 0.009 | 0.008 |
| Bond angles (º) | 1.033 | 0.946 |
| **Average B factors (Å^2^)** |  |  |
| Protein / water | 54.4 / 50.0 | 61.9 / 58.9 |
| Ligand atoms / ions | 54.4 / 78.5 | 48.0 / 83.1 |
| **Ramachandran plot** |  |  |
| Favoured / Allowed / Outliers (%) | 98.7 / 1.0 / 0.3 | 98.4 / 1.3 / 0.3 |
